# Successful Management of Thyrocervical Trunk Aneurysm Ruptured into the Thoracic Cavity After Cesarean Section in Nonstable Patient with Neurofibromatosis Type I

**DOI:** 10.3390/medicina61010049

**Published:** 2024-12-31

**Authors:** Nikola Mirković, Marko Prokić, Marija Novčić, Miloš Arsenijević, Snežana Sretenović, Dragan Knežević, Vojin Kovačević, Marija Šorak, Olivera Kostić

**Affiliations:** 1 Vascular Surgery Center, University Clinical Center Kragujevac, 34000 Kragujevac, Serbia; 2 Department of Surgery, Faculty of Medical Science, University of Kragujevac, 34000 Kragujevac, Serbia; 3 Department of Interventional Radiology, Department of Radiological Diagnostics, University Clinical Center Kragujevac, 34000 Kragujevac, Serbia; 4 Thoracic Surgery Center, University Clinical Center Kragujevac, 34000 Kragujevac, Serbia; 5 Clinic of Hematology, University Clinical Center Kragujevac, 34000 Kragujevac, Serbia; 6 Department of Internal Medicine, Faculty of Medical Science, University of Kragujevac, 34000 Kragujevac, Serbia; 7 Neurosurgery Center, University Clinical Center Kragujevac, 34000 Kragujevac, Serbia; 8 Gynecology and Obstetrics Clinic, Center for Biomedically Assisted Fertilisation, University Clinical Center Kragujevac, 34000 Kragujevac, Serbia; 9 Department of Gynecology and Obstetrics, Faculty of Medical Sciences, University of Kragujevac, 34000 Kragujevac, Serbia; 10 Department of Pharmacy, Faculty of Medical Sciences, University of Kragujevac, 34000 Kragujevac, Serbia; 11 Center for Research on Harmful Effects of Biological and Chemical Hazards, Faculty of Medical Sciences, University of Kragujevac, 34000 Kragujevac, Serbia

**Keywords:** thyrocervical trunk aneurysm, rupture, neurofibromatosis type I, puerperium, coil embolisation

## Abstract

Rupture of the thyrocervical trunk aneurysm into the thoracic cavity does not occur very often. It is an urgent condition due to hemorrhagic shock by massive hemothorax with potentially fatal consequences. Pregnancy and puerperium are additional risk factors for a rupture of the thyrocervical trunk aneurysm in patients with neurofibromatosis and aneurysms. This is the first case of thyrocervical trunk aneurysm rupture after a Cesarean section in a patient with neurofibromatosis type I noted down in the literature. The patient, a 33-year-old woman with neurofibromatosis type I, three days after an already performed Cesarean section had acute pain in the left area of the neck, swelling, and a hematoma that progressed rapidly to respiratory distress, hemothorax, and hemorrhagic shock. Emergency endotracheal intubation was performed for airway control. Urgent computer tomography angiography procedure showed extracranial artery, thyrocervical trunk aneurysm rupture, and vertebral aneurysm without rupture. The patient was urgently and successfully treated by endovascular coil embolization of a ruptured thyrocervical trunk aneurysm and subsequently thoracic drainage for massive hemothorax. Postoperatively, her left neck pain decreased, after which she had no further neurologic deficits. The patient was discharged 10 days later. Thyrocervical trunk aneurysm rupture is a rare condition with a potential outcome of death which requires urgent intervention. Endovascular coil embolization is a minimally invasive, safe, and efficient treatment for patients with rupture of thyrocervical trunk aneurysm and following comorbidities.

## 1. Introduction

Neurofibromatosis type 1 (NF-1), or von Recklinghausen’s disease, is an autosomal-dominant genetic disease that does not occur often and happens due to an abnormality of chromosome 17 [1,2,3]. The cause of NF-1 is the germline mutation of the tumor suppressor NF-1 gene. The NF-1 gene is located on chromosome 17q11.2, which encodes a cytoplasmic protein, neurofibromin, responsible for the inactivation of the RAS proto-oncogene, which is also responsible for cell growth. The mutation in the NF-1 gene leads to the activation of the RAS oncogene and the development of neurofibroma [4,5].

Specific for NF-1 are skin tumors, neurofibroma of multiple organs, dermatologic manifestations, and rarely vascular abnormalities [5,6,7]. Specific manifestations are pathognomonic Lisch nodules, café-au-lait spots, cutaneous neurofibroma, freckling and hyperpigmentation in the axillary or inguinal areas, optic glioma, pigmented iris hamartoma, osseous abnormalities, Schwann cell tumors, macrocephaly, intracranial tumors, neurologic impairment, kyphoscoliosis, osteopenia, syringomyelia, pheochromocytoma, and vasculopathy can occur among people with NF-1 [1,2,3]. Vasculopathy has been attributed to the impaired function of the NF-1 gene in the vascular endothelial cells, which leads to the proliferation and growth of the vascular endothelium. This causes fragility of the vessel wall and results in aneurysm formation and rupture [4].

Vasculopathy usually affects the arterial system. NF-1-related vasculopathy leads to aneurysms, stenoses, occlusions, aortic coarctation, arteriovenous malformations, and arterial compression or invasion by neural tumors [4,8,9,10,11]. Extracranial vascular abnormalities are unusual manifestations of NF-1. Extracranial vertebral artery aneurysm (VAA) occurs rarely, but the thyrocervical trunk aneurysm (TTA) is extremely rare in comparison. Patients with extracranial VAA or TTA typically present with radiculopathy, neck pain, or a neck mass, and—in those with rupture cervical hematoma, hemothorax and hypotension. VAA or TTA rupture can be a fatal condition if not recognized promptly and managed aggressively, especially when the patient presents with hemorrhagic shock. Very rarely, the aneurysm can hemorrhage into the thoracic cavity, leading to haemothorax [1,10,12,13]. Non-traumatic causes of extracranial aneurysms are usually associated with hypertension, infection, inflammatory disease, Ehler–Danlos syndrome, Marfan disease, Loeys–Dietz syndrome, and NF-1. These disorders are related to vasculopathy, including arterial aneurysms because they involve components of the extracellular matrix, which also compromises the integrity of vessel walls [12].

Criteria for operative or endovascular treatment were the presence of symptoms, rupture, or large aneurysm size. The same indications are being used in treatment for the repair of aneurysms in patients without neurofibromatosis. Vascular lesions that were asymptomatic and aneurysms of small diameter were observed [11,14,15]. Spontaneous extracranial aneurysms in patients with NF-1 occur most commonly in the third decade of life and are more common among the female population [12]. Primary extracranial TTA and VAA related to NF-1 is an extremely rare state and potentially a danger to a patient’s life because of possible rupture and is not well described due to its rare occurrence [10,12]. TTA rupture typically induces symptoms such as cervical pain, cervical hematoma, hemothorax, and hemorrhagic shock [10]. Rupture of TTA into the thoracic cavity is extremely uncommon, with potential death cases due to hemorrhagic shock by massive hemothorax that progressed rapidly to respiratory distress. Intrathoracic hemorrhage, although rare, should be considered one of the major and dangerous complications of VAA or TTA rupture with NF-1 [16,17]. Hemothorax in this condition, caused by rupture in a patient with NF-1 is an extremely rare condition, but it can be fatal. Rupture of the true TTA does not happen very often and very few cases have been reported up to date [4].

## 2. Case Description

We report the first case of ruptured TTA in a patient with NF-1 following a Cesarean section. A 33-year-old woman, three days after a Cesarean section presented with acute left neck pain, swelling, hematoma, and hypotension at another hospital, which progressed rapidly to respiratory distress and hemothorax. Emergency endotracheal intubation was performed because of difficulty when breathing and for airway control. She was then emergently transferred to our institution with the entourage of an anesthesiologist with the control of vital parameters. Past medical history was positive for NF-1 manifested by dermal and spinal neurofibromas, café-au-lait spots, and kyphoscoliosis of the cervical and thoracic spines. Multiple café au lait spots were visible in areas such as her chest and abdomen. The patient was previously operated on for scoliosis of the spine. She was also surgically treated for the sudden onset of lumbosacral hematoma pain and impaired mobility 4 years ago when resection of the neurofibroma in the lumbosacral region and evacuation of the hematoma was performed. Afterward, the skin defect was covered by a Thiersch skin graft. There was no history of injury or operation in the left supraclavicular region, clavicle fracture, or insertion of a central venous catheter. There was no history of tobacco use. The patient did not consume alcohol. She denies any sort of allergy. Her family history was significant. The mother is also suffering from NF-1.

**Figure 1:**
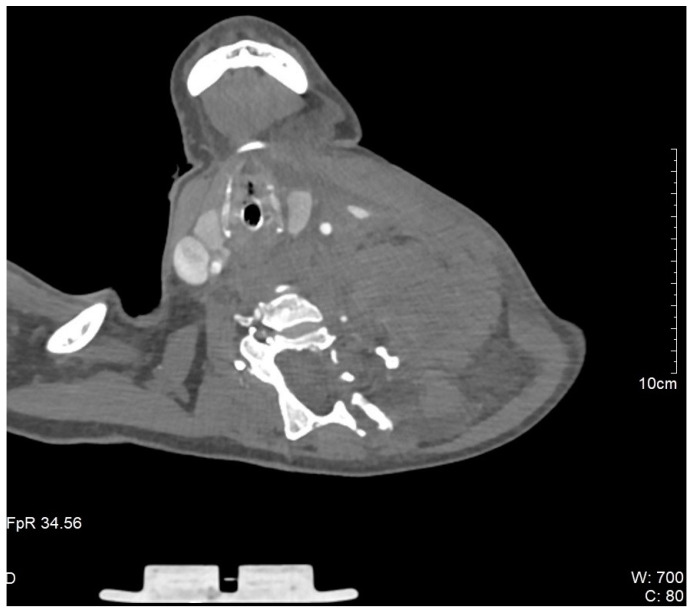
Chest computer tomography: Large left neck hematoma secondary to ruptured thyrocervical trunk aneurysm with compression on the surrounding organs.

**Figure 2:**
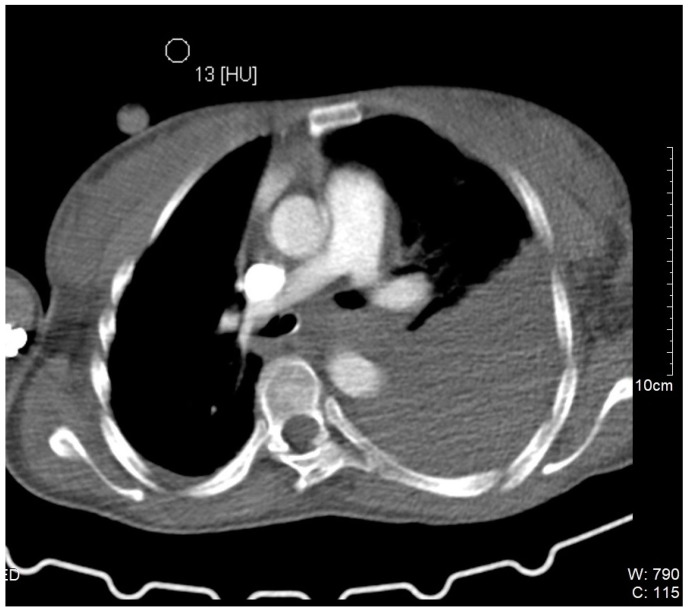
Chest computer tomography: Left hemothorax secondary to ruptured thyrocervical trunk aneurysm.

**Figure 3:**
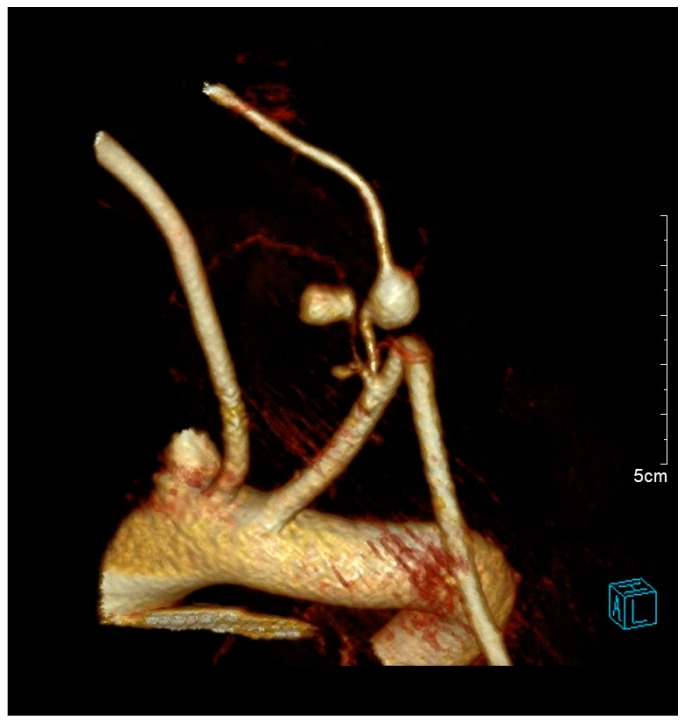
Computer tomography angiography of the extracranial artery: revealed a suspected ruptured aneurysm of the left thyrocervical trunk associated with active extravasation of contrast, and vertebral artery aneurysm without extravasation of contrast.

**Figure 4:**
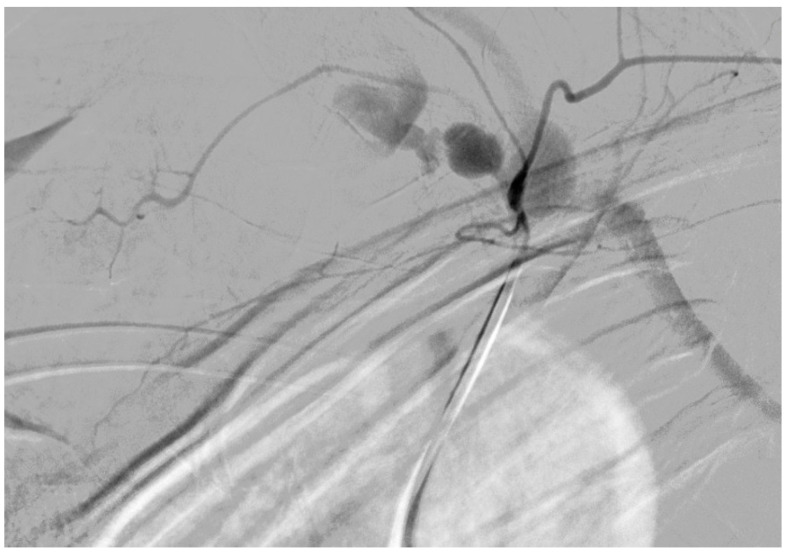
Digital subtraction angiography revealed a rupture of the left thyrocervical trunk aneurysm, with active extravasation of contrast and vertebral artery aneurysm without extravasation.

**Figure 5:**
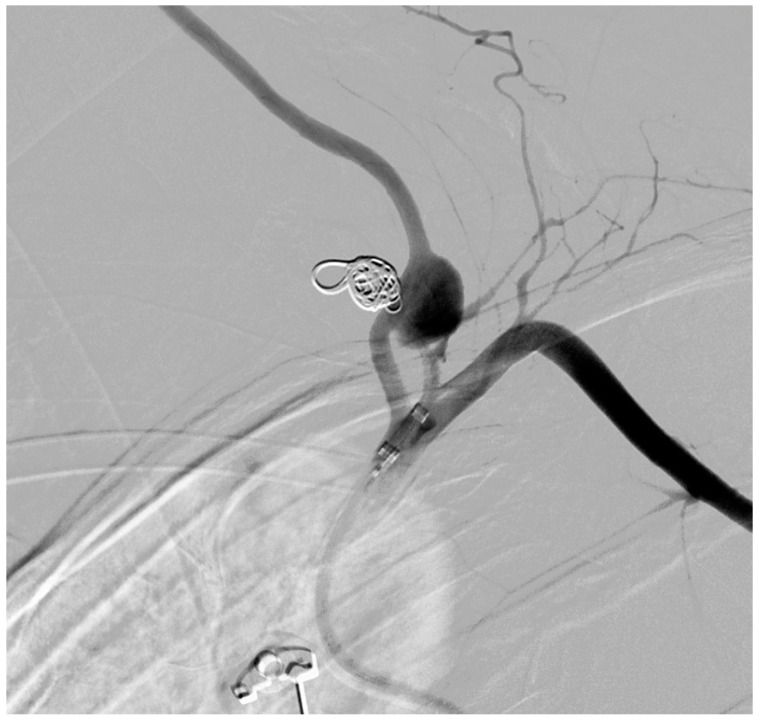
Postembolization arteriography revealed successful complete occlusion of the ruptured left thyrocervical trunk aneurysm by coil embolization.

**Figure 6:**
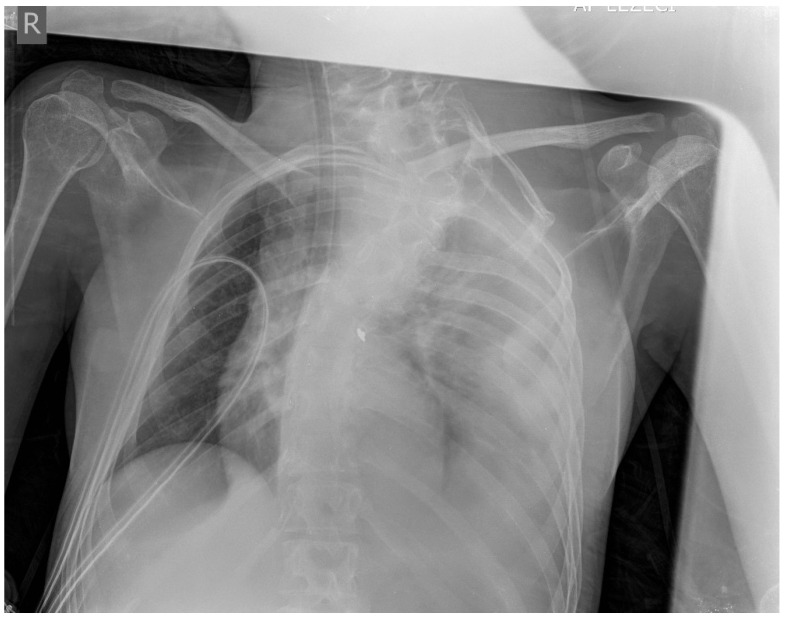
Frontal chest radiography demonstrates complete re-expansion of the lungs.

During the hospital admission, the patient was in a serious condition, with pain in the neck area, visibly scared and concerned. Physical examination was unremarkable except for a soft tissue swelling in the left supraclavicular fossa. Peripheral pulses were easily palpable in the referent places. Cranial nerves II–XII were intact, without neurological issues or damages. At the admission, she was tachycardic, tachypneic, hypotensive, and dyspneic. The admission hemoglobin levels and hematocrit levels were 9.9 g/dL and 30.4%, respectively. The patient had a blood pressure of 85/65 mmHg at the time of onset of the neck swelling. Intensive resuscitation of the patient was undertaken by the anesthesiologist. The patient was then transferred to the surgical intensive care unit for control of vital parameters, and stabilization of the condition, following reanimation and diagnostic procedure. It has been decided there was a need for an urgent computerized tomography scan procedure. The contrast-enhanced computerized tomography scan of the neck and chest revealed a left neck mass measuring 7.5 cm × 7 cm with compression of surrounding organs as well (Figure 1) and enormous left hemothorax (Figure 2). On the computer tomography angiography, the extracranial artery probably TTA has been shown with suspicion of rupture (Figure 3). After that digital subtraction angiography of extracranial artery was performed to find the precise and exact source of bleeding. An emergency digital subtraction angiography through a femoral puncture angiogram revealed a rupture of the left TTA, with active extravasation of contrast and VAA without extravasation (Figure 4). We have decided to perform urgent endovascular operative treatment of the ruptured aneurysm TTA. The decision was made considering the patient’s well-being, poor general condition, and following comorbidities. The patient was successfully treated by endovascular coil arterial embolization of a ruptured TTA. Postembolization arteriography showed satisfying results, completely occluding TTA (Figure 5). For the massive hemothorax on the left side, a chest tube was inserted through the fifth intercostal space on the medial axillary line and about 900 milliliters of blood was removed from the left pleural cavity. Her pulse rate decreased from a high 140 beats/min to 85 beats/min after the procedure. The placement of a chest tube for relief of the left hemothorax was deferred until the source of hemorrhage could be controlled. Her left neck swelling gradually decreased. Two days after embolization, when the patient was able to breathe without the endotracheal tube, it was removed without any further issues. Chest radiography was performed again and showed that in 5 days a complete expansion of the lungs occurred (Figure 6). The patient was returned to a regular diet and discharged from the hospital 10 days later. Postoperatively, her left neck pain improved, and she was discharged without further neurologic deficits.

## 3. Discussion

Vascular abnormalities that are associated with NF-1 are very rare and reportedly occur in a small number of cases. Neurofibromin, the protein product of the NF-1 gene, functions in part as a negative regulator of the p21 RAS proto-oncogene. The loss of neurofibromin expression results in increased mitogenic signaling and thus increased cell growth, which in turn facilitates tumor formation. Vascular abnormalities associated with NF-1 may reflect disruption of the vascular maintenance and repair regulated by neurofibromin [10]. Most of the patients with vascular abnormalities are asymptomatic. Symptoms usually occur in childhood or early adulthood. Renal artery lesions are the commonest followed by cerebrovascular lesions. Also, from the total number of NF-1-related vascular abnormalities the extracranial vessels are extremely rare conditions [4]. Although cases of extracranial VAA with NF-1 have previously been reported in the literature, rupture of an aneurysm into the thoracic cavity is reported in only a few of those, with a high mortality rate [10,16]. Also, this is one of the rare findings of rupture of left TTA complicated with hemothorax with mother in early puerperium. As for pregnant women, the risk of aortic dissection or rupture is elevated during pregnancy and the postpartum period [18]. Compared with non-pregnant and non-puerperal women, pregnant and puerperal women are faced with a significantly higher risk of vascular events [19]. TTA rupture can be a fatal condition if not recognized in time and managed aggressively, especially when the patient presents with hemorrhagic shock. Management options include operative surgical or endovascular treatment. Surgical treatment carries a higher risk of exsanguination, and long operation, with many complications and a possible death outcome in many cases. Operative treatment with endovascular coiling is necessary for the prevention of further complications and potentially fatal hemorrhage, as this method of treatment is minimally invasive and shorter in duration. Very rarely, the aneurysm can hemorrhage into the thoracic cavity, leading to hemothorax [12]. Ishizu reported a ruptured TTA in a patient with NF-1 and proposed that an arterial rupture occurs as a consequence of physiological factors in the setting of a fragile vascular wall [9]. Al-Jundi successfully managed a ruptured TTA by endovascular coiling. They preferred endovascular management in view of a previous complicated neck surgery [4,9,20]. The Pub-Med search engine of world literature discovered the first reported case of a patient with a ruptured true aneurysm of the TTA with hemothorax in a puerperium patient with NF-1. The importance of this case report is that there are no papers in the literature about TTA during pregnancy or after childbirth in a patient with NF-1 who was successfully treated with endovascular coiling of ruptured aneurysm with chest drainage. The patient had no complications of postoperative treatment. Sometimes embolization is not successful and is a necessary open surgical approach [21], but luckily this was not the case. The endovascular approach seems to be the most promising, but treatment needs to be adjusted to each individual patient [6]. Coil embolization can be added expeditiously in an elective or urgent setting, avoiding the risk of nerve injury (including Horner syndrome and vocal cord paralysis) reported with surgical resection. Surgical resection remains a definitive solution, especially in urgent conditions. This case demonstrates that endovascular surgery is better than open surgery because it is less invasive [22]. A careful examination aimed at early detection and treatment is required for this urgent condition. Endovascular coiling was safe even for an unstable patient with massive bleeding [15]. Patients with NF-1 who report neck pain, back pain, radiculopathy, cervical hematoma, or hemothorax should be suspected of having VAA, TTA, or intercostal artery aneurysm. There might be a necessity for routine screening for patients with NF-1 for vascular malformations of the extracranial arteries [23]. TTA is rare, but the mortality of ruptured cases is extremely high, so early diagnosis and early treatment are important. Endovascular treatment was very effective and safe [24]. The association between neurofibromatosis and vascular aneurysms is an often unrecognized but documented phenomenon. We would like to raise awareness of this infrequent presentation, as it is associated with high mortality and may be prevented by an early diagnosis [25].

In patients with neurofibromatosis, we should examine the following comorbidities and especially examine the possible abnormalities concerning the cardiovascular system. During pregnancy, there is an additional factor of risk appearing for an aneurysmal disease and rupture. In cases of female patients with neurofibromatosis who plan pregnancy, it is necessary to examine in detail all risk factors and inform the patient about possible complications that could occur and appear during pregnancy and in puerperium as well.

## 4. Conclusions

Rupture of TTA is a rare event that can have serious complications, consequences, and possibly fatal results. Hemothorax caused by TTA rupture in a patient with NF-1 in puerperium is an extremely rare condition. In these patients, an adequate and quick diagnosis based on the clinical symptoms and comorbidities, and urgent and effective treatment is the basis of successful treatment of this life-endangering state. Endovascular coil embolization is a minimally invasive, viable, effective, and safe method even for an unstable patient with massive bleeding. Careful examination aimed at early detection of TTA rupture and urgent treatment is required for this life-endangering condition.

Regarding all these facts, it is necessary that patients with neurofibromatosis should be examined in detail, paying attention to all possible abnormalities of cardiovascular system, comorbidities, and risks, especially in pregnant women since there are additional risk factors for the appearance of aneurysmal disease and rupture.

## Data Availability

Data is contained within the article.
